# Morality of the Heart: Heart Rate Variability and Moral Rule Adherence in Men

**DOI:** 10.3389/fnins.2021.612712

**Published:** 2021-09-07

**Authors:** Alexander Lischke, Matthias Weippert, Anett Mau-Moeller, Rike Pahnke

**Affiliations:** ^1^Department of Psychology, Medical School Hamburg, Hamburg, Germany; ^2^Department of Psychology, University of Greifswald, Greifswald, Germany; ^3^Department of Sport Science, University of Rostock, Rostock, Germany

**Keywords:** moral reasoning, moral decision making, idealism, harm avoidance, vagal tone

## Abstract

Moral rules are a cornerstone of many societies. Most moral rules are concerned with the welfare of other individuals, reflecting individuals’ innate aversion against harming other individuals. Harming others is associated with aversive experiences, implying that individuals who are sensitive to the aversiveness of these experiences are more likely to follow moral rules than individuals who are insensitive to the aversiveness of these experiences. Individuals’ sensitivity for aversive experiences depends on individuals’ ability to integrate the underlying neural and physiological processes: Individuals who are more efficient in integrating these processes are more sensitive to the aversiveness that is associated with moral rule violations than individuals who are less efficient in integrating these processes. Individuals who differ in their ability to integrate these processes may, thus, also differ in their inclination to follow moral rules. We tested this assumption in a sample of healthy individuals (67 males) who completed measures of moral rule adherence and integration abilities. Moral rule adherence was assessed with self-report measure and integration abilities were assessed with a resting state measure of heart rate variability (HRV), which reflects prefrontal–(para-)limbic engagement during the integration of physical and neural processes. We found a positive association between individuals’ HRV and individuals’ moral rule adherence, implying that individuals with efficient integration abilities were more inclined to follow moral rules than individuals with inefficient integration abilities. Our findings support the assumption that individuals with different integration abilities also differ in moral rule adherence, presumably because of differences in aversiveness sensitivity.

## Introduction

Across industrialized and non-industrialized societies, individuals share a common set of moral rules. Most moral rules are concerned with the welfare of others (Haidt and Joseph, 2004), which may be a reflection of individuals’ innate aversion against harming others (Hamlin et al., 2007, 2010). Regardless whether individuals imagine or perform harmful actions, they experience aversive emotional reactions that are accompanied by corresponding processes on the neural and physiological level (Cushman et al., 2012; Decety et al., 2012). The aversiveness of these experiences depends on individuals’ ability to integrate the corresponding neural and physiological processes (Critchley, 2005). Individuals who are efficient in integrating these processes are more sensitive to the aversiveness of harmful actions than individuals who are inefficient in integrating these processes (Cushman et al., 2012; Decety et al., 2012). As a consequence, individuals with efficient integration abilities are more motivated to avoid aversive experiences that are associated with moral rule violations than individuals with inefficient integration abilities (Decety and Cowell, 2014), implying more moral rule adherence in individuals with efficient than inefficient integration abilities.

Individuals’ integration abilities can be differentiated on the basis of individuals’ heart rate variability (HRV), which serves as a proxy for the interplay of prefrontal and (para-)limbic brain regions during the integration of neural and physiological processes (Smith et al., 2017). Individuals with higher HRV are generally more efficient in integrating these processes than individuals with lower HRV, implying that individuals with higher HRV are also more efficient in integrating neural and physiological processes that are associated with aversive experiences. These differences in integration abilities may render individuals with higher HRV more sensitive to the aversiveness of others’ harm than individuals with lower HRV. Individuals with higher HRV are, in fact, more responsive to others’ needs and more concerned with others’ welfare than individuals with lower HRV (Kogan et al., 2014; Stellar et al., 2015; Lischke et al., 2018a), presumably because individuals with higher HRV are more empathetic and less alexithymic than individuals with lower HRV (Lischke et al., 2017, Lischke et al., 2018b). Due to these differences in aversiveness sensitivity, individuals with higher HRV may be more inclined to follow moral rules than individuals with lower HRV.

Following this notion, we performed an exploratory study where we investigated the association between HRV and moral rule adherence in a sample of healthy individuals. In the absence of previous studies on HRV and moral rule adherence, we felt obliged to provide a concise description rather than a complex explanation of the association between HRV and moral rule adherence. We, thus, tested whether HRV was associated with moral rule adherence and refrained from testing whether this association would be moderated or mediated by aversiveness sensitivity. This allowed us to analyze the association between HRV and moral rule adherence in a clear and simple manner, thereby avoiding issues arising from the use of more complex analyses (e.g., overfitting in structural equation models).

As we were interested to investigate the association between HRV and moral rule adherence in a clear and simple manner, we made arrangements to reduce the complexity of the study design. Given that male and female individuals differ in moral rule adherence and HRV (Abhishekh et al., 2013; Friesdorf et al., 2015), we only included male individuals in our investigation. We, thus, did not have to control for sex- or menstrual cycle-related differences in moral rule adherence and HRV in our analyses. To reduce the number of possible analyses to a minimum, we used a limited set of measures for the assessment of moral rule adherence and HRV. HRV was assessed with a resting state measure of high-frequency HRV (HF-HRV). HF-HRV measures the integration of neurophysiological processes that are associated with an empathic reaction to others’ harm (Kogan et al., 2014; Stellar et al., 2015; Lischke et al., 2018b), implying that this may also be the case during violations of moral rules that are concerned with others’ welfare. Moral rule adherence was assessed with a self-report measure that differentiated between moral idealism and moral relativism (Forsyth, 1980). Whereas moral idealism is characterized by strict rule following that precludes the violation of moral rules, moral relativism is characterized by flexible rule following that allows the violation of moral rules (Forsyth, 1980). Given that moral idealism reflects moral rule following to a greater extent than moral relativism, we expected individuals’ HRV to be associated with individuals’ moral idealism rather than with individuals’ moral relativism.

## Materials and Methods

### Participants

According to an *a priori* power analysis with G^∗^Power^1^, we had to investigate a minimum of 67 individuals to be able to detect medium- to large-sized associations between HRV and moral rule adherence in a series of dimensional and categorical analyses [1−β = 0.80, α = 0.05, *f*^2^ = 0.20, and *f* = 0.35]. In order to be included in the study, individuals had to be males with an age range of 18 to 35 years. Individuals who were in psychotherapeutic or psychopharmacological treatment were excluded from the study. Inclusion and exclusion of individuals was determined on the basis of an in-house interview that assessed individuals’ demographic (age and sex), anthropometric (height and weight), and health (physical activity, psychotherapeutic treatment, and psychopharmacological treatment) characteristics. Of the 67 individuals who had been recruited for the study, 3 individuals had to be excluded because they were in psychotherapeutic treatment. The final sample, thus, comprised 64 instead of 67 individuals (see Table 1). However, all individuals had provided written informed consent to the study protocol that was approved by the ethics committee of the University of Rostock and that was carried out in accordance with the Declaration of Helsinki.

**TABLE 1:** Sample characteristics.

	*M*	SEM
Age (years)	23.91	0.49
Body mass index (kg/m^2^)	23.76	0.28
Activity (h/week)	7.66	0.49
Moral relativism (EPQ-IDE)	5.98	0.11
Moral idealism (EPQ-REL)	5.68	0.14
Heart rate variability (HF-HRV, ms^2^)^*a*^	2.73	0.05
Heart rate variability (RMSSD, ms)^*a*^	1.59	0.02

*EPQ-REL, Ethical Position Questionnaire—Moral relativism (Forsyth, 1980); EPQ-IDE, Ethical Position Questionnaire—Moral idealism (Forsyth, 1980); HF-HRV, (log-transformed) high-frequency heart rate variability (Shaffer and Ginsberg, 2017); and RMSSD, (log-transformed) root mean square of successive differences between consecutive heart beats (Shaffer and Ginsberg, 2017).*

*^*a*^ Data were missing for one participant due to a recording error.*

### Procedure

We followed an established procedure that has been described in more detail elsewhere (Lischke et al., 2017, Lischke et al., 2018a,b). At the beginning of the experimental session, individuals were asked to use the bathroom to control for the effects of bladder filling and gastric distension on individuals’ HRV. Thereafter, individuals were seated in a comfortable chair and prepared for a 5-min lasting resting state heart rate (HR) recording. Individuals’ HR was recorded with a polar watch (RS800, Polar Electro, Oy, Kempele, Finland) that allowed an accurate assessment of consecutive changes in heartbeats. The consecutive changes in heartbeats were later used for the determination of individuals’ HRV. During the HR recording, individuals were instructed to sit still, to breathe spontaneously, and to keep their eyes open. After the HR recording, individuals had to complete a self-report measure of moral rule adherence (Ethical Position Questionnaire, EPQ; Forsyth, 1980). At the end of the experimental session, individuals were debriefed and dismissed.

### Heart Rate Variability

Kubios HRV 2.2 (Tarvainen et al., 2014) was used to determine individuals’ HRV on the basis of the HR recordings. The HR recordings were detrended (smoothn priors: λ = 500) and, if necessary, artifact corrected (adaptive filtering: cubic spine interpolation) before they were subjected to a spectral analysis (Fast Fourier Transformation) and a time domain analysis. The spectral analysis was used to determine the HRV measure of interest: HF-HRV (0.15–0.4 Hz). HF-HRV was the HRV measure of interest because HF-HRV tracks the integration of neurophysiological processes that are associated with an empathic reaction to others’ harm (Kogan et al., 2014; Stellar et al., 2015; Lischke et al., 2018b), indicating that HF-HRV reflects aversive reactions to violations of others’ welfare (see Supplementary Material 1). The time domain analysis was used for the determination of a HRV measure that tracks similar processes as HF-HRV (Shaffer and Ginsberg, 2017): the root mean square of successive differences between consecutive heart beats (RMSSD). This HRV measure was used to determine whether possible associations between HF-HRV and moral rule adherence would generalize across different HRV measures.

### Moral Rule Adherence

The EPQ (Forsyth, 1980) was used to determine individuals’ moral rule adherence in terms of moral idealism and moral relativism. The self-report measure comprises 20 items, 10 items that assess moral idealism [α = 0.64] and 10 items that assess moral relativism [α = 0.73]. Whereas moral idealism refers to moral rule adherence in terms of strict rule following that precludes the violation of moral rules in all circumstances, moral relativism refers to moral rule adherence in terms of flexible rule following that allows the violation of moral rules in some circumstances. Given that moral rule adherence is motivated by the concern about others’ welfare (Haidt and Joseph, 2004), it is not surprising that moral idealism (i.e., strict rule following) rather than moral relativism (i.e., flexible rule following) is associated with aversive reactions to violations of others’ welfare (see Supplementary Material 2).

### Statistical Analysis

In line with recent recommendations (Laborde et al., 2017), dimensional and categorical analyses were performed to investigate the association between HRV and moral rule adherence in the sample of individuals. Combining dimensional and categorical analyses allowed a cross-validation of the respective findings, thereby providing a robustness check for any conclusions that were based on the findings of a particular analysis. For the dimensional analysis, hierarchical regression analyses were run to investigate whether HRV was associated with moral rule adherence among all individuals. For the categorical analyses, analyses of covariance (ANCOVAs) were run to investigate whether moral rule adherence differed between individuals who had been assigned to a high and low HRV group on the basis of a median-split. For both types of analyses, HRV was log-transformed (log 10) to account for deviations from normality. Age, body mass index, and physical activity were under statistical control in these analyses because these characteristics may affect the association between individuals’ HRV and individuals’ moral rule adherence (De Meersman, 1993; Abhishekh et al., 2013; Koenig et al., 2014). To facilitate the interpretation of the analyses (Cohen, 1992; Cumming, 2014), significance values (*p*) and effect size measures (η^2^, *B*, *R*^2^, and Δ*R*^2^) were determined. All analyses were performed with SPSS 24 (SPSS Inc., Chicago, IL, United States).

## Results

### Association Between Moral Relativism (EPQ-REL) and Heart Rate Variability (HF-HRV)

**FIGURE 1:**
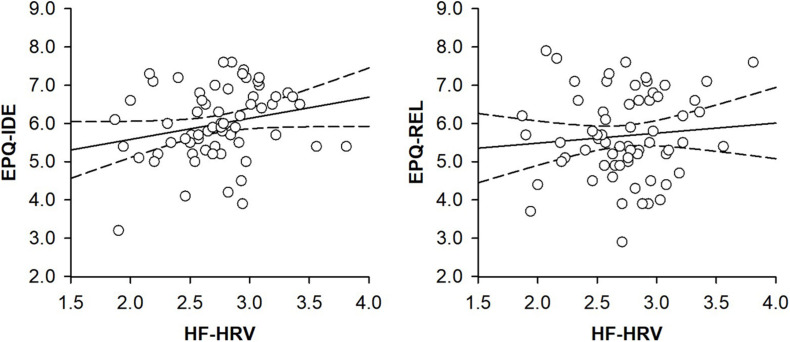
Scatter plots with lines of best fit and 95% confidence intervals demonstrating associations between (log-transformed) heart rate variability (HF-HRV) and moral idealism (EPQ-IDE) or moral relativism (EPQ-REL) among all individuals.

**FIGURE 2:**
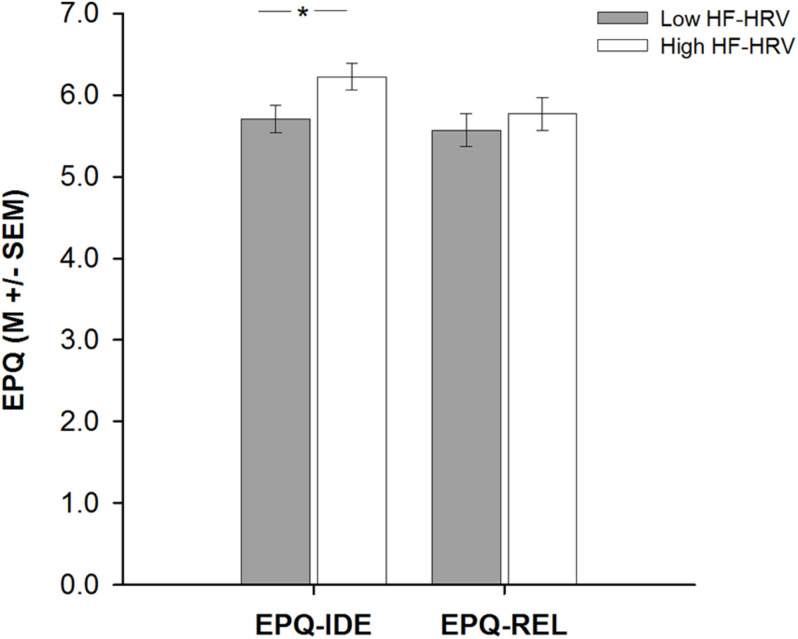
Bar plots demonstrating differences in moral idealism (EPQ-IDE) or moral relativism (EPQ-REL) between individuals with lower and higher heart rate variability (HF-HRV). Bars represent M ± SEM. *p ≤ 0.05.

A hierarchical regression analysis was run to investigate the association between HF-HRV and moral relativism among all individuals. Entering individuals’ age, body mass index, and physical activity in a first step into the regression model did not explain any variance in individuals’ moral relativism [*R*^2^ = 0.02, *F*(3, 59) = 0.49, and *p* = 0.693; see Table 2]. Age, body mass index, and physical activity were not associated with moral relativism [all *B* ≤ | 0.05|, all *t*(59) ≤ | 1.20|, and all *p* ≥ 0.233; see Table 2]. Entering individuals’ HF-HRV in a second step into the regression model also explained no variance in moral relativism [Δ*R*^2^ = 0.01, Δ*F*(1, 58) = 0.38, and *p* = 0.538; see Table 2]. HF-HRV was, similar to age, body mass index, and physical activity [all *B* ≤ | 0.04|, all *t*(58) ≤ | 1.07|, and all *p* ≥ 0.287; see Table 2], not associated with moral relativism [*B* = 0.24, *t*(58) = 0.62, and *p* = 0.538; see Table 2 and Figure 1]. A subsequent ANCOVA revealed that moral relativism was equally pronounced among individuals with higher and lower HF-HRV [*F*(1,58) = 0.31, *p* = 0.581, and η^2^ = 0.005; see Figure 2], thereby confirming the absence of an association between individuals’ HF-HRV and individuals’ moral relativism. Repeating the analyses with RMSSD instead of HF-HRV revealed exactly the same findings (see Supplementary Material 3), indicating that the findings generalize across different HRV measures (HF-HRV, RMSSD).

**TABLE 2:** Association of moral idealism (EPQ-IDE) or moral relativism (EPQ-REL) with heart rate variability (HF-HRV).

	Moral idealism (EPQ-IDE)						Moral relativism (EPQ-REL)		
Model 1	*B*	SE B	*t*	*p*	Model 2	*B*	SE B	*t*	*p*
***Step 1***					***Step 1***				
Age (years)	−0.01	0.04	−0.34	0.738	Age (years)	0.05	0.04	1.20	0.233
Body mass index (kg/m^2^)	0.00	0.05	−0.07	0.947	Body mass index (kg/m^2^)	−0.04	0.08	−0.54	0.595
Activity (h/week)	−0.01	0.03	−0.39	0.699	Activity (h/week)	0.01	0.04	0.29	0.776
*Step 2*					*Step 2*				
Age (years)	−0.02	0.04	−0.70	0.486	Age (years)	0.04	0.04	1.07	0.287
Body mass index (kg/m^2^)	0.03	0.05	0.45	0.655	Body mass index (kg/m^2^)	−0.03	0.08	−0.37	0.717
Activity (h/week)	−0.03	0.03	−0.95	0.347	Activity (h/week)	0.00	0.04	0.11	0.913
Heart rate variability (HF-HRV, ms^2^)^*a*^	0.66	0.33	2.12	0.039*	Heart rate variability (HF-HRV, ms^2^)^*a*^	0.24	0.44	0.62	0.538

*Model 1: step 1: *R*^2^ = 0.01, *F*(3, 59) = 0.10, *p* = 0.959, step 2: Δ*R*^2^ = 0.07, Δ*F*(1, 58) = 4.48, *p* = 0.039^∗^; model 2: step 1: *R*^2^ = 0.02, *F*(3, 59) = 0.49, *p* = 0.693, step 2: Δ*R*^2^ = 0.01, Δ*F*(1, 58) = 0.38, *p* = 0.538.*

*EPQ-REL, Ethical Position Questionnaire—Moral relativism (Forsyth, 1980); EPQ-IDE, Ethical Position Questionnaire—Moral idealism (Forsyth, 1980); and HF-HRV, (log transformed) high-frequency heart rate variability (Shaffer and Ginsberg, 2017).*

*^*a*^Data were missing for one participant due to a recording error.*

*^∗^*p**

*≤ 0.05.*

### Association Between Moral Idealism (EPQ-IDE) and Heart Rate Variability (HF-HRV)

Another hierarchical regression analysis was run to investigate the association between HF-HRV and moral idealism among all individuals. Entering individuals’ age, body mass index, and physical activity in a first step into the regression model explained no variance in individuals’ moral idealism [*R*^2^ = 0.01, *F*(3, 59) = 0.10, and *p* = 0.959; see Table 2]. Age, body mass index and physical activity were not associated with moral idealism [all *B* ≤ | − 0.01|, all *t*(59) ≤ | − 0.39|, and all *p* ≥ 0.699; see Table 2]. Entering individuals’ HF-HRV in a second step into the regression model explained 7% of the variance in individuals’ moral idealism [Δ*R*^2^ = 0.07, Δ*F*(1, 58) = 4.48, and *p* = 0.039; see Table 2]. Whereas age, body mass index, and physical activity remained to be unassociated with moral idealism [all *B* ≤ | − 0.03|, all *t*(58) ≤ | − 0.95|, and all *p* ≥ 0.347; see Table 2], HF-HRV turned out to be associated with moral idealism [*B* = 0.66, *t*(58) = 2.12, and *p* = 0.039; see Table 2 and Figure 1]. A subsequent ANCOVA showed that moral idealism was more pronounced among individuals with higher than lower HF-HRV [*F*(1,58) = 5.11, *p* = 0.028, and η^2^ = 0.081, see Figure 2], thereby confirming the existence of a positive association between individuals’ HF-HRV and individuals’ moral idealism. The positive association between individuals’ HF-HRV and individuals’ moral idealism remained unchanged when controlling for individuals’ moral realism in the analyses [Δ*R*^2^ = 0.07, Δ*F*(1, 57) = 4.16, *p* = 0.046; *B* = 0.65, *t*(57) = 2.04, and *p* = 0.046; see Table 3]. Repeating the analyses with RMSSD instead of HF-HRV revealed similar findings (see Supplementary Material 4), indicating that the findings generalize across different HRV measures (HF-HRV, RMSSD).

**TABLE 3:** Association of moral idealism (EPQ-IDE) with heart rate variability (HF-HRV) under control of moral realism (EPQ-REL).

	Moral Idealism (EPQ-IDE)			
Model 1	*B*	SE B	*t*	*p*
*Step 1*				
Age (years)	−0.02	0.04	−0.47	0.640
Body mass index (kg/m^2^)	0.00	0.06	0.00	0.997
Activity (h/week)	−0.01	0.03	−0.42	0.675
Moral relativism (EPQ-REL)	0.10	0.11	0.90	0.373
*Step 2*				
Age (years)	−0.03	0.04	−0.80	0.428
Body mass index (kg/m^2^)	0.03	0.06	0.48	0.631
Activity (h/week)	−0.03	0.04	−0.96	0.343
Moral relativism (EPQ-REL)	0.08	0.11	0.75	0.454
Heart rate variability (HF-HRV, ms^2^)^*a*^	0.65	0.35	2.04	0.046^∗^

*Note. Model 1: step 1: *R*^2^ = 0.02, *F*(3, 58) = 0.28, *p* = 0.892, step 2: Δ*R*^2^ = 0.07, Δ*F*(1, 57) = 4.16, *p* = 0.046^∗^.*

*EPQ-REL, Ethical Position Questionnaire—Moral relativism (Forsyth, 1980); EPQ-IDE, Ethical Position Questionnaire—Moral idealism (Forsyth, 1980); and HF-HRV, (log-transformed) high-frequency heart rate variability (Shaffer and Ginsberg, 2017).*

*^*a*^Data were missing for one participant due to a recording error.*

*^∗^*p* ≤ 0.05.*

## Discussion

In the present study, we investigated whether individuals’ ability to integrate neural and physiological processes was associated with individuals’ tendency to follow moral rules. Individuals’ integration abilities were determined on the basis of a HRV measure that served as a proxy for the interplay of prefrontal and (para-)limbic brain regions during the regulation of neural and physiological processes (Smith et al., 2017). Individuals’ tendency to follow moral rules was assessed with a self-report measure that differentiated between moral idealism and moral relativism (Forsyth, 1980). Applying these measures to a sample of healthy individuals, we found an association between HRV and moral idealism but no association between HRV and moral relativism: moral idealism was more pronounced among individuals with higher than lower HRV, whereas moral relativism was equally pronounced among individuals with higher and lower HRV. These findings emerged in a series of complementary analyses, which helped to ascertain the robustness of the observed associations. To understand these associations, we have to consider that moral idealism and moral relativism refer to distinct but overlapping aspects of moral rule following. Whereas moral idealism refers to strict rule following that precludes the violation of moral rules in all circumstances, moral relativism refers to flexible rule following that allows the violation of moral rules in some circumstances. We considered the conceptual overlap of moral idealism and moral relativism in our analyses and still found an association between moral idealism and HRV. Our findings, thus, show that individuals with higher HRV follow moral rules to a greater extent (i.e., in all circumstances) than individuals with lower HRV. Given that differences in HRV reflect differences in neurophysiological integration (Smith et al., 2017), our findings support the assumption that individuals with higher integration abilities show more moral rule adherence than individuals with lower integration abilities.

Whether individuals with efficient and inefficient integration abilities follow moral rules may depend on their sensitivity for aversive experiences that are associated with real or imagined violations of moral rules (Cushman et al., 2012; Decety et al., 2012). Individuals whose psychological traits render them aversiveness sensitive, like, for example, empathetic individuals (Conway and Gawronski, 2013; Reynolds and Conway, 2018), are more inclined to follow moral rules than individuals whose psychological traits render them aversiveness insensitive, like, for example, alexithymic individuals (Koven, 2011; Patil and Silani, 2014). These differences in moral rule adherence are even more pronounced in individuals who show abnormal representations of these psychological traits, like, for, example, autistic or psychopathic individuals (Koenigs et al., 2012; Patil et al., 2016). However, individuals who differ in empathy, alexithymia, autism, or psychopathy also seem to differ in their ability to integrate neural and physiological processes as suggested by the respective differences in individuals’ HRV (Hansen et al., 2007; Kuiper et al., 2017; Lischke et al., 2018b). It may, thus, be possible that differences in individuals’ integration abilities contribute to differences in individuals’ aversiveness sensitivity that lead to differences in individuals’ moral rule adherence. Considering that individuals’ HRV reflect differences in individuals’ integration abilities (Smith et al., 2017), we assume that individuals with lower HRV showed more moral rule adherence than individuals with higher HRV because of differences in individuals’ aversiveness sensitivity for moral rule violations.

Individuals with efficient integration abilities are more successful in engaging prefrontal and (para-)limbic brain regions for the regulation of neural and physiological processes than individuals with inefficient integration abilities (Critchley, 2005), indicating that differences in prefrontal–(para-)limbic engagement may account for differences in aversiveness sensitivity between individuals with efficient and inefficient integration abilities. However, differences in prefrontal–(para-)limbic engagement may account not only for differences in individuals’ aversiveness sensitivity but also for differences in individuals’ moral rule adherence because naturally occurring or experimentally induced alterations in these brain regions impair individuals’ integration abilities as well as individuals’ moral rule adherence (Mendez and Shapira, 2009; Moretto et al., 2010; Tassy et al., 2012). Individuals with abnormal representations of empathy, alexithymia, autism, and psychopathy also show alterations in prefrontal and (para-)limbic brain regions that are associated with impairments in aversiveness sensitivity and moral rule adherence (Silani et al., 2008; Decety et al., 2013), indicating that overlapping networks of prefrontal and (para-)limbic brain regions are implicated in the integration of neural and physiological processes that are relevant for the experience of aversiveness in the context of moral rule violations (Decety and Cowell, 2014). The interplay of prefrontal and (para-)limbic brain regions can be assessed on the basis of individuals’ HRV (Smith et al., 2017), suggesting that differences in individuals’ HRV reflect differences in individuals’ integration abilities that are due to differences in prefrontal–(para-)limbic engagement. We, thus, assume that individuals with higher HRV showed more moral rule adherence than individuals with lower HRV because individuals with higher HRV were more efficient in engaging prefrontal and (para-)limbic brain regions for the integration of neurophysiological processes that account for the aversiveness of moral rule violations than individuals with lower HRV.

Although these assumptions appear to be plausible, we think that the assumptions have to be validated in further studies that follow a less exploratory approach than the present study. These studies should investigate the association between individuals’ HRV and individuals’ moral rule adherence with more complex measures and in more diverse samples than the present study. The present study investigated this association in a homogenous sample of male individuals, leaving open whether similar associations would emerge in heterogeneous samples that include male and female individuals. Given that female individuals have a higher HRV and a higher moral rule adherence than male individuals (Abhishekh et al., 2013; Friesdorf et al., 2015), the association between HRV and moral rule adherence may be more pronounced among female than male individuals. Future studies that include male and female individuals may help to determine whether this is the case. These studies should also include male and female individuals with a more diverse background in their investigation to determine whether the proposed associations between individuals’ HRV and individuals’ moral rule adherence generalize across different populations (e.g., including individuals with different ages or ethnicities). The present study explored this association with a combination of physiological and self-report measures. Although these measures allowed us to describe the association between HRV and moral rule adherence in a concise manner, they did not allow us to provide a complex explanation of this association. Neural, physiological, and behavioral measures that may have helped to provide such an explanation were not employed (e.g., task-based measures of moral rule adherence and aversiveness sensitivity and imaging-based measures of neurophysiological integration). We were, thus, unable to probe the psychological and neurobiological mechanisms underlying the association between HRV and moral rule adherence (e.g., testing the mediating or moderating role of aversiveness sensitivity). These mechanisms may involve the neurophysiological integration of aversive reactions to violations of others’ welfare, but whether this is in fact the case remains to be determined. Studies that combine neural, physiological, and behavioral measures in their investigation may be more successful in elucidating the psychological and neurobiological mechanisms of HRV and moral rule adherence than the present study (e.g., combining task-based measures of moral rule adherence and aversiveness sensitivity with imaging-based measures of neurophysiological integration). Studies that manipulate these mechanisms with appropriate methods may help to make causal inferences about the association between HRV and moral rule adherence (e.g., increasing or decreasing HRV with brain stimulation techniques), thereby providing first insights into intervention programs for individuals who have difficulties in moral rule following (e.g., HRV biofeedback training for individuals with psychopathy). We hope that our exploratory study opened an avenue for these types of studies.

To sum up, we found a positive association between HRV and moral rule adherence in a sample of healthy individuals. Individuals with higher HRV showed more moral rule adherence than individuals with lower HRV. We assume that the differences in individuals’ moral rule adherence were due to differences in individuals’ aversiveness sensitivity for moral rule violations that were determined by differences in individuals’ prefrontal–(para-)limbic engagement during the integration of neurophysiological processes. As we based these assumptions on the findings of previous studies (Decety and Cowell, 2014; Smith et al., 2017), we encourage researchers to validate these assumptions in further studies. Together, these studies will provide important insights into the psychological and neurobiological mechanisms underlying the association between individuals’ HRV and individuals’ moral rule adherence. Whether these studies will help to develop treatment interventions for individuals who have difficulties in moral rule adherences has to be seen in the future.

## Data Availability Statement

The datasets presented in this article are not readily available because of ethical restrictions. Requests to access the datasets should be directed to AL.

## Ethics Statement

The studies involving human participants were reviewed and approved by University of Rostock. The patients/participants provided their written informed consent to participate in this study.

## Author Contributions

AL and RP designed the study, analyzed the data, and wrote the manuscript. AL, AM-M, and MW collected the data. AM-M and MW contributed to writing, reviewing, and editing of the manuscript. All authors approved the final version of the manuscript.

## Conflict of Interest

The authors declare that the research was conducted in the absence of any commercial or financial relationships that could be construed as a potential conflict of interest.

## Publisher’s Note

All claims expressed in this article are solely those of the authors and do not necessarily represent those of their affiliated organizations, or those of the publisher, the editors and the reviewers. Any product that may be evaluated in this article, or claim that may be made by its manufacturer, is not guaranteed or endorsed by the publisher.

## Footnotes

1
http://www.gpower.hhu.de
