# Supplementary material for: Morality of the Heart: Heart Rate Variability and Moral Rule Adherence in Men
Source: Front Neurosci. 2021 Sep 7;15:612712. doi: 10.3389/fnins.2021.612712 (PMC8452936; doi:10.3389/fnins.2021.612712)
Supplement: Supplementary file 1 [file Data_Sheet_1.pdf]

Morality of the Heart: Heart Rate Variability and Moral Rule Adherence in Men

Alexander Lischke[1,2], PhD; Matthias Weippert, PhD[3]; Anett Mau-Moeller[3], PhD & Rike Pahnke[3]

[1]Department of Psychology, Medical School Hamburg, Hamburg, Germany

[2]Department of Psychology, University of Greifswald, Greifswald, Germany

[3]Department of Sport Science, University of Rostock, Rostock, Germany

Supplemental Methods

S1 Association between heartrate variability and aversion against violations of others' welfare

High frequency heart rate variability (HF-HRV) tracks the integration of neurophysiological processes that are associated with the responsiveness to others' need and the compassion for others' harm (Kogan et al., 2014; Lischke et al., 2018; Stellar, Cohen, Oveis, & Keltner, 2015). We, thus, assumed that HF-HRV would be associated with the concern about others' welfare.

To test this assumption, we administered the empathetic concern scale[1] of the Interpersonal Reactivity Index (IRI; Davis, 1983) to a separate sample of individuals (n = 53) whose heart rate had been recorded under resting state conditions (5 min). We used Kubios HRV 2.2 (Tarvainen, Niskanen, Lipponen, Ranta-Aho, & Karjalainen, 2014) to obtain HRF-HRV and another HRV measure, the root mean square of successive differences between consecutive heart beats (RMSSD), from the heart rate recordings. Correlation analyses (controlling for age, body mass index and activity) revealed correlations of empathic concern with HF-HRV [$r(48) = .301$, $p = .034$] and RMSSD [$r(48) = .244$, $p = .088$]. The correlation coefficients suggest that HF-HRV rather than RMSSD is associated with an empathic aversion against violations of others' welfare. HF-HRV, thus, tracks the integration of empathic processes that motivate moral rule adherence in the context of harm aversion (Conway & Gawronski, 2013; Gleichgerrcht & Young, 2013; Patil & Silani, 2014; Reynolds & Conway, 2018).

[1] We decided to operationalize the concern for others' welfare in terms of empathic concern because empathic concern refers to an evolutionary acquired motivation to protect others' from harm and to feel compassion for others' harm (Decety & Cowell, 2014).

S2 Association between moral rule adherence and aversion against violations of others' welfare

Moral idealism and moral relativism represent different aspects of moral rule adherence, which is motivated by the concern about others' welfare (Haidt & Joseph, 2004). The concern about others' welfare should, thus, be differentially associated with moral idealism and moral relativism. Moral idealism refers to moral rule adherence in terms of strict rule following that precludes the violation of moral rule, whereas moral relativism refers to moral rule adherence in terms of flexible rule following that allows the violation of moral rules. Given that moral idealism represents moral rule adherence in a narrower sense than moral relativism, we assumed that moral idealism would be closer associated with the concern about others' welfare than moral relativism.

We tested this assumption in a separate sample of individuals (n = 38) who completed the moral idealism and moral relativism scale of the Ethical Positions Questionnaire (EPQ; Forsyth, 1980) alongside the empathetic concern scale[2] of the IRI (Davis, 1983). A correlation analyses revealed a correlation between empathic concern and moral idealism [$r(33) = .580$, $p \leq .001$] but no correlation between empathic concern and moral relativism [$r(33) = .223$, $p = .212$; $z = 1.851$, $p = .032$, $q = .425$], indicating that moral idealism is associated with an empathic aversion against violations of others' welfare. Moral idealism, thus, shares the same motivational basis as other forms of morality that have been related to an innate aversion of harming others (Conway & Gawronski, 2013; Gleichgerrcht & Young, 2013; Patil & Silani, 2014; Reynolds & Conway, 2018).

---

[2] We decided to operationalize the concern for others' welfare in terms of empathic concern because empathic concern for the same reasons that have been outlined in Footnote 1.

Supplemental Results

S3 Association between moral relativism (EPQ-REL) and heart rate variability (RMSSD)

A hierarchical regression analysis was run to investigate the association between RMSSD and moral relativism among all individuals. Entering individuals' age, body mass index and physical activity in a first step into the regression model, did not explain any variance in individuals' moral relativism [R^2 = .02, F(3, 59) = 0.49, p = .693; see Table S1]. Age, body mass index and physical activity were not associated with moral relativism [all $B \leq |0.05|$, all $t(59) \leq |1.20|$, all $p \geq .233$; see Table S1]. Entering individuals' HRV (RMSSD) in a second step into the regression model, also explained no variance in moral relativism [ΔR^2 = .02, ΔF(1, 58) = 0.49, p = .693; see Table 2]. RMSSD was, similar as age, body mass index and physical activity [all $B \leq |0.04|$, all $t(58) \leq |1.05|$, all $p \geq .298$; see Table S1], not associated with moral relativism [B = 0.55, t(58) = 0.69, p =.491; see Table S1]. A subsequent ANCOCA revealed that moral relativism was equally pronounced among individuals with higher and lower RMSSD [F(1,58) = 0.31, p = .567 , η^2 = 0.006; see Table S1], thereby confirming the absence of an association between individuals' RMSSD and individuals' moral relativism. Together, the aforementioned analyses revealed exact the same findings as the analyses that used HF-HRV instead of RMSSD as the HRV measure of interest.

S4 Association between moral idealism (EPQ-IDE) and heart rate variability (RMSSD)

A hierarchical regression analysis was run to investigate the association between RMSSD and moral idealism among all individuals. Entering individuals' age, body mass index and physical activity in a first step into the regression model, explained no variance in individuals' moral idealism [R^2 = .01, F(3, 59) = 0.10, p = .959; see Table S1]. Age, body mass index and physical activity were not associated with moral idealism [all $B \leq |-0.01|$, all $t(59) \leq |-0.39|$, all $p \geq .699$; see Table S1]. Entering individuals' RMSSD in a second step into the

regression model, explained of 6% of the variance in individuals' moral idealism [$\Delta R^2 = .07$, $\Delta F(1, 58) = 4.48$, $p = .056$; see Table S1]. Whereas age, body mass index and physical activity remained to be unassociated with moral idealism [all $B \leq |-0.02|$, all $t(58) \leq |-1.06|$, all $p \geq .292$; see Table 2], RMSSD was, at least on a trend level, associated with moral idealism [$B = 1.27$, $t(58) = 1.94$, $p = .057$; see Table S1]. A subsequent ANCOVA showed that moral idealism was, at least on a trend level, more pronounced among individuals with higher than lower RMSSD [$F(1,58) = 3.39$, $p = .071$, $\eta^2 = 0.055$; see Table S1], thereby confirming the existence of a positive association between individuals' RMSSD and individuals' moral idealism. Overall, the analyses revealed similar findings as the analyses that used HF-HRV instead of RMSSD as the HRV measure of interest.

Supplemental Tables

Table S1

Association of moral idealism (EPQ-IDE) or moral relativism (EPQ-REL) with heart rate variability (RMSSD)

	Moral idealism (EPQ-IDE)					Moral relativism (EPQ-REL)			
Model One	*B*	*SE B*	*t*	*p*	Model two	*B*	*SE B*	*t*	*p*
Step one					*Step one*				
Age (years)	-0.01	0.04	-0.34	.738	Age (years)	0.05	0.04	1.20	.233
Body mass index (kg/m^2)	0.00	0.05	-0.07	.947	Body mass index (kg/m^2)	-0.04	0.08	-0.54	.595
Activity (h/w)	-0.01	0.03	-0.39	.699	Activity (h/w)	0.01	0.04	0.29	.776
Step two					*Step two*				
Age (years)	-0.02	0.04	-0.70	.490	Age (years)	0.04	0.04	1.05	.298
Body mass index (kg/m^2)	0.02	0.06	0.39	.699	Body mass index (kg/m^2)	-0.03	0.08	-0.36	.724
Activity (h/w)	-0.04	0.04	-1.06	.292	Activity (h/w)	0.00	0.04	0.02	.984
Heart rate variability (RMSSD, ms)[a]	1.27	0.81	1.94	.057†	Heart rate variability (RMSSD, ms)[a]	0.55	0.78	0.69	.491

Note. Model one: step one: $R^2 = .01$, $F(3, 59) = 0.10$, $p = .959$, step two: $\Delta R^2 = .06$, $\Delta F(1, 58) = 3.78$, $p = .057^{\dagger}$, model two: step one: $R^2 = .02$, $F(3, 59) = 0.49$, $p = .693$, Step 2: $\Delta R^2 = .01$, $\Delta F(1, 58) = 0.48$, $p = .491$.
EPQ-REL = Ethical Position Questionnaire – Moral relativism (Forsyth, 1980), EPQ-IDE = Ethical Position Questionnaire – Moral idealism (Forsyth, 1980), RMSSD = (log transformed) log-transformed root mean square of successive differences between consecutive heart beats (Shaffer & Ginsberg, 2017).
[a] Data was missing for one participants due to a recording error.
† $p \leq .08$.

Supplemental References

Conway, P., & Gawronski, B. (2013). Deontological and utilitarian inclinations in moral decision making: a process dissociation approach. *J Pers Soc Psychol, 104*(2), 216-235.

Davis, M. (1983). Interpersonal Reactivity Index. A multidimensional approach to individual differences in empathy. *J Pers Soc Psychol, 44*, 113-126.

Decety, J., & Cowell, J. M. (2014). The complex relation between morality and empathy. *Trends Cogn Sci, 18*(7), 337-339.

Forsyth, D. R. (1980). A taxonomy of ethical ideologies. *J Pers Soc Psychol, 39*(1), 175.

Gleichgerrcht, E., & Young, L. (2013). Low levels of empathic concern predict utilitarian moral judgment. *PLoS One, 8*(4), e60418.

Haidt, J., & Joseph, C. (2004). Intuitive ethics: How innately prepared intuitions generate culturally variable virtues. *Daedalus, 133*(4), 55-66.

Kogan, A., Oveis, C., Carr, E. W., Gruber, J., Mauss, I. B., Shallcross, A., . . . Keltner, D. (2014). Vagal activity is quadratically related to prosocial traits, prosocial emotions, and observer perceptions of prosociality. *J Pers Soc Psychol, 107*(6), 1051-1063.

Lischke, A., Pahnke, R., Mau-Moeller, A., Behrens, M., Grabe, H. J., Freyberger, H. J., . . . Weippert, M. (2018). Inter-individual differences in heart rate variability are associated with inter-individual differences in empathy and alexithymia. *Front Psychol, 9*, 229.

Patil, I., & Silani, G. (2014). Reduced empathic concern leads to utilitarian moral judgments in trait alexithymia. *Front Psychol, 5*, 501.

Reynolds, C. J., & Conway, P. (2018). Not just bad actions: Affective concern for bad outcomes contributes to moral condemnation of harm in moral dilemmas. *Emotion, 18*(7), 1009-1023.

Shaffer, F., & Ginsberg, J. P. (2017). An overview of heart rate variability metrics and norms. *Front Public Health, 5*, 258.

Stellar, J. E., Cohen, A., Oveis, C., & Keltner, D. (2015). Affective and physiological responses to the suffering of others: compassion and vagal activity. *J Pers Soc Psychol, 108*(4), 572-585.

Tarvainen, M. P., Niskanen, J. P., Lipponen, J. A., Ranta-Aho, P. O., & Karjalainen, P. A. (2014). Kubios HRV--heart rate variability analysis software. *Comput Methods Programs Biomed, 113*(1), 210-220.
